# The impact of doctorate-timing, whether undergraduate or postgraduate, on the experience of German doctors

**DOI:** 10.1186/s12909-026-08849-w

**Published:** 2026-02-19

**Authors:** Ammar Al Naimi, Anna Hentrich, Franz Bahlmann, Frank Louwen

**Affiliations:** 1 Goethe University, Frankfurt, Germany; 2 Department of Obstetrics and Gynecology, Buergerhospital - Dr. Senckenberg Foundation, Nibelungenallee 37-41, 60318 Frankfurt, Germany

**Keywords:** German doctorate, Dr.med., Undergraduate research, Postgraduate research

## Abstract

**Background:**

German medical students obtain a licence to practice medicine without being awarded an academic title. They can acquire the Doctor of Medicine title upon finishing a doctoral thesis that can be undertaken as undergraduates or later as postgraduates. This study aims to investigate how and if the timing of a German doctorate affects the candidates ‘ experience.

**Methods:**

This is a qualitative study based on in-depth semi-structured interviews to analyse the perception of German doctors of their own doctoral projects. The ontology of this work is relativism with a subjective epistemology and empirical phenomenology as a methodology. Partly inductive, partly deductive reflexive thematic analysis according to Braun and Clarke was utilized.

**Results:**

We identified four themes which were motivations, struggles, support and outcomes. Both undergraduate and postgraduate doctorates are identity-driven, whereas scientific curiosity and ambition are undergraduate motivations. Inadequate supervision, unpreparedness, and personal commitments are shared struggles for all doctorates. The sources of support for both undergraduate and postgraduate doctorates are similar, and the positive impact of doctorates outweighs the negative regardless of timing.

**Conclusions:**

Postgraduate doctorates are neither inherently better nor worse than undergraduate doctorates. Nevertheless, the timing of the doctorate seems to affect the experience of researchers. Developing a professional identity is the unanimous motivation for doctorates among German medics, but undergraduates are additionally motivated by ambition and scientific curiosity. Difficulties facing doctoral candidates are not directly related to the timing of research relative to graduation, rather to their developmental stage. Mentors, family, peers and research groups are essential sources of support. These findings provide insight into interesting themes that should be further explored.

**Clinical trial number:**

Not applicable.

**Supplementary Information:**

The online version contains supplementary material available at 10.1186/s12909-026-08849-w.

## Introduction

German medical students obtain a licence to practice medicine upon finishing their final exam without being awarded an academic degree with a title [1]. These graduates use the professional title of a ‘physician’, “Arzt”, instead of the academic title of a ‘doctor’ that is otherwise awarded to medical graduates worldwide. German physicians can acquire the Doctor of Medicine, “Dr. med.”, title upon conducting a research project and successfully defending the corresponding doctoral thesis [2]. The majority of German physicians pursue a doctorate in order to acquire this title despite the fact that it is an academic achievement that is not required for practising medicine [3]. A doctorate and a title could boost prestige and self-image, improve employability, promote continuing education, and facilitate the acquisition of knowledge and skills [2].

These doctorates can be undertaken by medical students as undergraduates or by physicians at any later point during their career as postgraduates. Regardless of when the doctorate is conducted, the title can only be granted after graduation. While around 15% of medical students show no interest in research and accordingly do not undertake a doctorate [2], the majority of students initiate a doctorate during medical school, although only three quarters of them indeed finish their projects [4]. Nevertheless, the ratio of abandoned doctorates is high at around 15% by undergraduates during medical school and about 21% of students forsake at least one doctorate [5, 6]. A cross-sectional study from 2016 showed a high drop-out rate where 25% of supervisors reported a total of 208 discontinued projects, and around 20–30% of doctoral projects do not finish [7]. Moreover, despite the lack of statistical significance, there was a trend for increasing rates of abandoned doctorates from 21 to 26% from 2001 to 2011 [8]. The proportion of abandoned German doctorates in medicine (21%) is especially important because it is almost twice as high as those in biology, chemistry and veterinary medicine [9].

The burden caused by deserted doctorates on the educational system is immense due to the waste of effort and money spent on these projects, it is therefore desirable to assist researchers in overcoming obstacles and finishing their doctorates [10]. These projects are not mere methods of acquiring an academic title, but they are believed to significantly affect development and shape the clinical identity. They encourage reflection, promote research competencies, entice scientific curiosity, elevate critical thinking, and advance teamwork [11–14].

A qualified supervisor is named for every German doctoral candidate at the beginning of their project to assist them throughout their research. The research question is usually developed by the supervisor who secures workplace and resources. More importantly, high quality supervision is an essential factor in ensuring successful doctorates and German universities have adopted doctoral contracts as a mean for regulating and maintaining the supervisory relationships [15].

Suboptimal supervision combined with personal circumstances, increasing workload, time constraints, waning motivation and methodological incompetency are the main causes of abandoning or not starting doctorates [16]. Doctorates could prolong medical school by increasing the study-/workload by more than fifty percent and the acquisition of research competencies could compete with that of clinical knowledge for available time, thus negatively impacting academic grades [17]. These findings raise the question of whether it might be better to limit doctoral research to the time of postgraduate speciality training to improve the researcher’s experience and decrease the rate of deserted projects. Data suggest that starting doctorates late during medical school is associated with better thesis outcomes and higher success rates [18]. It is unclear, though, whether this apparent effect of time could have been caused by the gradual accumulation of essential skills for successful doctorates or a change in study load with fewer time constraints at later stages of medical school [18].

To date, there is no educational research investigating the differences between undergraduate and postgraduate German doctorates. Therefore, it is of utmost importance to identify substantially different outcomes in students’ perceptions and projects’ results in order to inform and ideally optimise future practices. The aim of this study is to investigate if and how the timing of undertaking a German doctorate differentially affects the candidates ‘ experience and outcomes.

## Methods

This is a qualitative study based on in-depth semi-structured interviews which analyses the perception of German doctors of their own doctorate research projects. The ontology of this work is relativism with a subjective epistemology and empirical phenomenology as a methodology [19]. Phenomenology places the experience from a first-person view at the centre of analysis as it recognises the value of this experience which usually cannot be assessed purely with quantitative methods [20].

Blind-copied group-email invitations, which were approved by the data protection board, were sent to all working physicians at two teaching German hospitals. This invitation asked eligible candidates, physicians with a Dr. med. title, to voluntarily participate in 45- to 60-min-long interviews, either face-to-face or virtually. The sample size was set to a total of eight with four participants within each of two cohorts: doctorates conducted during medical school or during specialty training. Conducting more interviews was an option if additional data for meaningful analysis were deemed necessary, but the study concluded without the need for revising the initial sample size due to the high quality and abundance of collected data [21]. Convenience sampling was utilised for pragmatic reasons and the first 4 volunteer physicians from each cohort were scheduled for the interviews.

The interviews were based on a framework-driven, pre-conceived interview guide developed for this study and provided as a supplement (S1). All interviews were conducted in native German language. The guiding framework for this project and the interviews relied on andragogy and Grow’s staged self-directed learning (SSDL). The assumptions of andragogy describe the learners as adults with a crystallised self-image and who are mindful of the benefit of learning. Thus, they voluntarily initiate their learning experience with self-directedness [22]. They are ready to learn and benefit from their background learning experience which enriches their engagement with learning. Moreover, their internal motivation is a driving force for acquiring knowledge and skills that they can successfully apply to their daily activities [23]. We assume that doctorates are the perfect example of andragogical practice [24]. Therefore, these overarching assumptions and principles are the backbone underlying this project to identify differences in motivation, engagement, and involvement depending on time of conduct. Postgraduate and undergraduate doctoral students are supposedly chronologically at different developmental stages. Grow’s SSDL was devised as a model to assist teachers in guiding their students towards autonomy. This model includes four stages for both teachers and students, where higher stages are associated with increasing autonomy and decreasing need for support. According to Grow, it is essential for teachers to adapt and match students’ needs and stages for effective learning [25]. Therefore, mentorship, supervision, support and self-directedness are explored as additional venues of interest within the framework of this study and directly shape the interview guide. While the guide provided a common skeleton for the interviews, these were still iterative and certain aspects and domains that emerged in one interview were integrated into the structure of the subsequent ones.

The transcribed German interviews were analysed with partly inductive, partly deductive reflexive thematic analysis according to Braun and Clarke [26]. This method of analysis was chosen because of its suitability for understanding experiences, thoughts, and behaviours. The initial step of analysis involved data familiarisation which was achieved through listening to the interviews twice while proof-reading the transcripts for accuracy. Coding was directly derived from the assumptions of andragogy and the mentorship components of Grow’s SSDL model [22, 25]. Two rounds of coding resulted in seven broad categories that cover the important concepts for testing the proposed theories [27]. These were Motivation and start, Concept, conduct and involvement, Difficulties, Help and support, Supervision and Guidance, Benefits and Losses. The codes were gathered into topics and data interpretation and integration led to provisional themes. Analysis resumed by searching for further themes within the coded data, reviewing and refining the resulting themes, and scaffolding them into more defined, and eventually renamed themes before the final theoretical concepts were presented.

The ethical approval for this study was obtained from the University of Frankfurt Ethics Committee (2022–574) and was subsequently approved by the Research Governance Manager at the School of Clinical Medicine of the University of Cambridge.

## Results

A total of eight interviews (three virtual and five face-to-face) were conducted, where the individual interviews lasted between 25 and 61 min, and the total recorded interview time to be transcribed was 5 h and 6 min. The doctorates were granted by five different German universities between 1988 and 2017. All four undergraduate doctorate participants finished the first project they started, whereas three from the postgraduate cohort abandoned at least one project they attempted during medical school. The experiences from these abandoned undergraduate doctorates were included within the analysis of this project. The speciality fields of the participants were ophthalmology, gynaecology, anaesthesiology, surgery, intensive care medicine and epidemiology. One out of four of the undergraduate cohort conducted their project in their future speciality field, while three out of four of the postgraduate cohorts performed projects within their speciality field. Some of the demographic information about the study participants is summarised in Table 1.

**Table 1:** A summary of the current position, gender, number of abandoned doctorates, and the year and the University of the granted doctorates for the study participants

Participant’s ID	Gender	Current position	Granting University	Number of abandoned doctorates	Date granted
UG1	Male	Head physician	Frankfurt	0	1990
UG2	Female	Head physician	Mainz	0	1988
UG3	Female	Consultant	Gießen	0	2015
UG4	Male	Head physician	Frankfurt	0	1995
PG1	Female	Senior consultant	Mainz	0	2017
PG2	Male	Senior consultant	Berlin	1	2003
PG3	Female	Senior consultant	Göttingen	2	2004
PG4	Female	Senior consultant	Frankfurt	2	2011

Four main themes were identified from the collected data:

### Theme I (Motivations—Both undergraduate and postgraduate doctorates are identity-driven, whereas scientific curiosity and ambition are undergraduate motivations)

Both cohorts provided a diverse list of reasons behind their decision to start a research project. The motivations were both intrinsic and extrinsic with personal and public benefit in mind. “Es gehört dazu” was a unanimous answer provided by everyone when asked about the motivation to undertake a doctorate which translates to “it is part of it”. The interviewees’ clarification of this statement included PG3 “I did not want to be called a doctor without having the title”, UG2 “I would not have taken myself seriously or respected myself without having the title”, UG3 “not having the title is embarrassing”, and PG1 “I did not want to pretend to be something that I am not……It always bothered me to be called a doctor by patients even though I was not one, and I got tired of explaining why a physician is not necessarily a doctor”. These statements could highlight the role of professional identity as a motivation. PG4 stated “Even though I felt like a physician, the title made me really feel complete … I felt like something was missing … and afterwards I felt complete”. PG2 said “I was fascinated by basic sciences and medical knowledge seemed like a journey of discovery into humankind … I enjoyed most of the courses which elevated my interest in conducting a doctorate … It is a shame that my fascination with scientific discovery during medical school was not properly utilised and supported for a doctorate”.

Both cohorts were extrinsically motivated to undertake doctorates, though these motivations slightly differed. Most undergraduates are suddenly faced with abundant free time after finishing the basic science semesters, and this gives them a reason to seek opportunities for investing that time. Students who believe that they should wisely utilise their time grasp this opportunity at this particular point in time and start considering projects that meet their educational and aspirational needs. Some students seek projects in a specific field of medicine because they see it as a future speciality field, but most students do not. They undertake any project that seemed interesting at the time or was somehow offered to them, without committing to that field as a future speciality. This might highlight their scientific curiosity as an intrinsic motivation. Undergraduates are career oriented, goal focused, and their doctorates are often attempts to secure a training position, a specific speciality, a research career, or a scholarship. Postgraduates are already in training positions and are more likely to choose doctorates within their field of daily practice. Their decision to initiate a doctorate is circumstantial, opportunistic, and based on convenience. Compatibility with their professional duties and time commitment is a leading reason to start a research project. Their familiarity with the subject, job arrangements such as part-time work, and theoretical integration into daily practice provides a construct within which they feel comfortable with their ability to achieve their goals PG1 “they made it very easy for me to do it", “I would not have done it if I had had to work in a laboratory” … PG3 “I would not have done it, had the opportunity not presented itself”. Only one of the postgraduate cohort participants mentioned career progression as their motivation in that they wanted to establish a new division within the department and needed the experience from the research project to acquire the qualifying skill set.

### Theme II (Struggles—Inadequate supervision, unpreparedness, and personal commitments are shared struggles for all doctorates)

There are numerous difficulties that face undergraduates and postgraduates during the course of their doctorates. Many of those are shared by both cohorts such as private obligations, inadequate supervision, and the lack of required skills. One of the undergraduates had to juggle school, research, work, spouse and three small children UG1 “it was very difficult for me with the three kids, and it was definitely difficult for them as well …. I lost my first marriage because of the doctorate because I was egocentric and prioritised my research instead of my spouse”. Another example shows a postgraduate who was single and had no family or financial commitments and therefore did not mention private obligations as a difficulty.

Inadequate supervision was a difficulty that faced both cohorts. Apart from one study participant, all interviewees reported that they had close to zero contact with their educational supervisors UG1 “The supervisor was almost never seen, he was God and the Pope … I knew him from the lectures, and he was catastrophic, he insulted students who questioned him, he was God and we were just trash”, PG3 “our scheduled appointments lasted less than three minutes … I felt unsupported … he failed to get goals and milestones for us”.

The lack of required skills was observed in both cohorts, but interestingly this was more prevalent and varied in the undergraduate cohort. The lacking skills for this cohort were required at all the stages of research from literature review to laboratory skills, IT-skills, up to thesis writing. The skills that the postgraduate cohort lacked were mainly laboratory skills and knowledge of statistics. Additional miscellaneous difficulties were experienced by both cohorts such as departmental politics UG4 “there was a miscommunication between my supervisor and the head of the laboratory” as well physical and psychological stress UG1 “it was the most stressful experience of my entire life … it was frustrating” for the undergraduates. One postgraduate had to deal with constant pressure and questions from funders as the project was funded by a pharmaceutical company and the researcher needed to produce results.

### Theme III (Support—The sources of support for both undergraduate and postgraduate doctorates are similar)

Support came from involved supervisors, peers and research groups, family and friends, as well as external sources such as research funders. Educational supervisors are supposed to have the leading roles in guiding, coaching and supporting junior researchers during their projects [28]. Mentors are senior researchers who could function as a proxy for the supervisors and provide the juniors with guidance and support. The mentors were shown to be instrumental in supporting researchers both undergraduate and postgraduate. Mentors guide the researchers, provide them with feedback, signpost them, identify their weaknesses and devise mitigations, and represent aspirational figures for the researchers UG2 “my mentor was the right one to have, he was always available and approachable, he was such a fine man … He was interested and wanted to be informed of what we were writing or publishing … The project was his baby … He supported us and we supported him … even though we never considered ourselves as his peers, he always treated us well and was a great role model”.

Three individuals from the postgraduate cohort had a mentor who was initially a close friend before they mentored the candidates. These mentors were often the catalyst for initiating the postgraduate doctorate. They approached the candidates and encouraged their motivations to pursue a doctorate with the promise of help and support that they genuinely delivered PG3 “I would have never started it if it was not my friend offering the opportunity … I would have never been able to finish without my friend, he motivated me, gave me literature to read, guided me through writing … he provided the theoretical design … I did not want to embarrass or disappoint my mentor and friend”.

The support of family members and friends was more significant for the undergraduate cohort. This help came in vastly different forms including financial support, technical help, encouragement, baby-sitting and homemaking. Two of the postgraduate cohort indeed mentioned that their partners gave them emotional support during the doctorate, but these statements pale in comparison to the passionate statements of the undergraduate cohort such as UG1 “extended family helped, and it was not for my mother-in-law then I would have never finished my doctorate” and UG4 “I married early, and my spouse could financially support me to pursue a poorly paid research career”. The external source of support one postgraduate candidate received came from research funders who helped with data analysis, statistics, and publishing the results.

Peers and research groups were instrumental in obtaining required skills, brainstorming and feedback, motivating each other, as well as maintaining morale for both cohorts. The research groups include medical technical assistants, peers working on related projects, peers working on other projects, junior researchers, senior researchers, and supervisors. Support can be provided at any of these levels UG3 “the technical assistants at the laboratory taught me the skills needed and they were accustomed to teaching these”, “we were a very good team that had a room to work together, and we taught and supported each other … we motivated each other within the team and became very close … motivated each other through writing the thesis”. One of the postgraduate study participants reflected on their previously abandoned project and named the lack of a research group as a reason for failing PG2 “I wish I were in a research group where each had their own project to meet, discuss, motivate and help each other”.

### Theme IV (Outcomes—The positive impact of doctorates outweighs the negative regardless of timing)

The undergraduate cohort reported a more diverse outcome profile both positive and negative in comparison to the postgraduate cohort. Undertaking and successfully finishing doctorates boosted the self-esteem of the undergraduate cohort, improved their ability to effectively communicate with others by promoting teamwork and even forging life-long lasting friendships to cherish PG3 “We learned teamwork as we needed to coordinate our experiments according to timing and availability of corresponding machines … one team member became a friend for life … I am to this day proud of what I have achieved every time I write the two letters of the title in front of my name”. In addition to the benefits on a personal level, the professional future of the undergraduate cohort was more significantly affected by their research experience than that of the postgraduate cohort. It improved their scientific knowledge of the field, and they were able to acquire several skills for conducting research such as designing projects, statistical analysis, writing proposals and laboratory techniques. These in turn fostered the ability to pursue an academic career for this cohort and increased the likelihood of future involvement with research. Furthermore, their employability improved, and they were able to obtain jobs more quickly regardless of whether their doctorates were topical for the chosen field or not. Moreover, those surveyed reported that their progression within their job was accelerated so that they reached higher levels more quickly than their peers without doctorates.

The professional life of the postgraduate cohort did not witness a benefit from the doctorate and their positions, duties, and progression did not change. They were able to attend conferences, give talks and presentations, and experience the outlines of a researcher’s career, but this experience did not result in a shift of direction towards academia or research as PG2 phrased it “I had a short detour into the world of science and conferences with workshops and talks”. They still valued the acquired knowledge from their doctorates as beneficial for expanding their horizons. The sense of pride that was demonstrated by the undergraduate cohort was mirrored by the postgraduate cohort as well.

The doctorates were associated with some negative outcomes. Undergraduates often required a free semester which provided them with protected research time. This prolonged their medical studies and they graduated later than their non-researcher peers. While it was possible for some students to conduct their research on top of their educational obligations without taking a free semester, this endeavour significantly affected their personal carefree student life and was associated with physical and psychological stress. This could be extreme enough to cause a post-traumatic distress that pushes the candidates aways from the field of research UG3 “I lost the joy of doing research for a long, long time … it remains a negatively remembered experience”. Postgraduates who faced lots of difficulties remember the doctorate as a generally negative experience, but not to the extent of the undergraduates.

## Discussion

It seems that the academic title is somehow linked to the physicians’ identity. The label of a doctor is a significant part of the physician’s identity as they are addressed with that title several times daily. Every single interaction with a patient is associated with denominating the physician as “doctor”, and it is exhausting and embarrassing to continuously correct patients and explain to them how German physicians should not be called a doctor without finishing a doctorate. One could argue that what patients call you is not important if they do not understand the difference, but apparently physicians care about that. The study participants felt the need to correct how they were addressed, and either did not want to take undeserved credit or felt embarrassed by justifying why they did not hold the title. This societal expectation seems to affect how physicians see themselves and could alter their sense of identity. They perceive the title as an integral part of the professional identity and do not feel complete without it. The medical professional identity is perceived by junior medical students as ‘given to’ or assigned by the society, but they develop and break out of this mould with reflection, interprofessional interactions and patient contact [29].

**Fig. 1:**
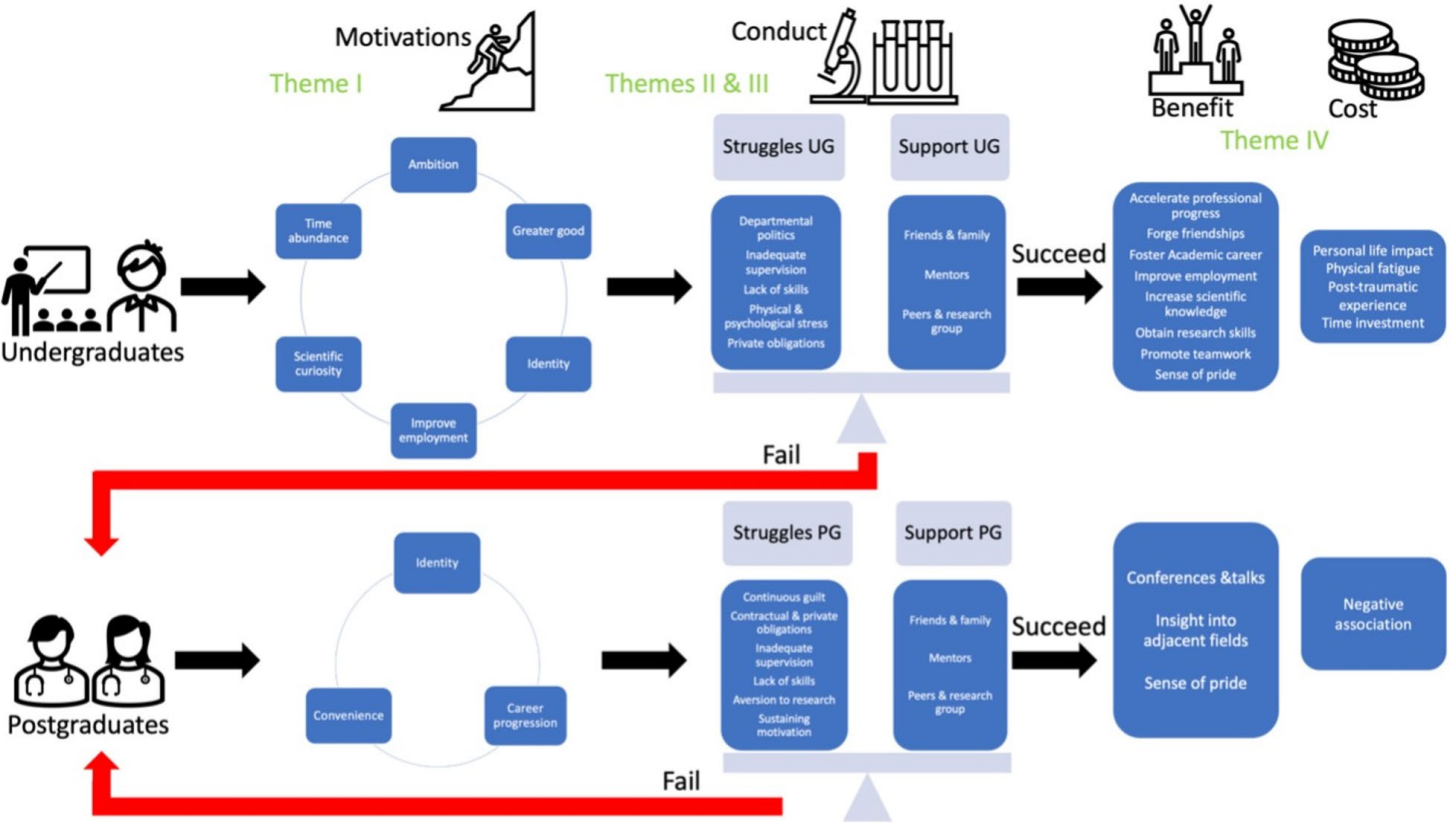
A flowchart of the German doctorate experience according to this study. It shows the relationship between abandoned and successful doctorates, and it summarizes the different motivations for both undergraduate and postgraduate candidates to undertake a doctoral research project, the struggles and support they encounter during the conduct, and their main outcomes after successfully finishing their projects

Not having the title plants a seed for self-doubt and insecurity, and this eventually buds into a need and internal motivation to undertake countermeasures by initiating a doctorate. While this claim should be based on interviewing physicians without a doctorate, it could also be appropriated from the experience of the abandoned doctorates in our cohort. A participant with Austrian roots shared that it was not an option for them not to have the title. It is automatically granted with the medical degree in their country of origin and while they studied medicine in Germany, they needed to feel equivalent to the Austrian graduates. This difference in education and academic titles could result in feelings of inferiority and eventually lead to an inferiority complex for medical graduates without a title in comparison to their national and international peers [30]. Identity is the maintained driving motivation for participants with abandoned undergraduate doctorates to undertake a second postgraduate project after previous failures. This relationship is shown as a red arrow in Fig. 1.

The undergraduate cohort exhibited other intrinsic motivations for starting a doctorate. They often described themselves as ambitious and required venues where they could vent and invest their potential. Research is perceived as their attempt to create something from the ground up to satisfy their ambition. They believe in themselves and their ability to change the world. Undergraduates want to truly leave a mark on medicine and see doctorates both as obligations and opportunities to achieve that goal. They genuinely want to help fellow human beings as per the Hippocratic Oath and seek to improve their medical knowledge and skills in order to fulfil that oath [31]. This striving for the greater good fuelled by their fascination with the various disciplines of medicine drives them to choose their individual research projects. This characteristic of naivety seems to get lost with time and was not observed in the postgraduate cohort. Graduating from medical school, undertaking the responsibility of working in a hospital, and starting speciality training all change an individual’s personality and worldview. This could be seen as the innate aspiration level that is quite high for young medical students longing to become a ‘doctor’. This does not indicate that they aspire to achieve a title instead of practicing medicine, rather to embody the ideal image of a practicing physician that is acknowledged with academic achievements. Increasing progress into a professional career and achieving the goal of becoming a practising physician decreases the aspiration level through subjective satisfaction with one’s own achievements [32]. While this change can be a natural part of growing up and maturing, it could also be considered a waste of time-dependent resources. Therefore, educators should desire to promote doctorates during medical school and encourage young medical students to implement and utilise their scientific curiosity before these qualities are lost to the coarseness of maturity.

Identity represents the most stable motivation for German physicians to perform research regardless of timing, whereas undergraduates display an array of additional motivations such as ambition and scientific curiosity. Nurturing and supporting these motivations with special emphasis on undergraduate doctorates rather than those of postgraduates could be the right direction for fostering future scientists and research enthusiasts that could move medicine forward. This does not necessarily mean that postgraduates are less motivated to undertake doctorates, rather their focus is shifted. Identity as a motivation and their zeal to feel complete could grow with time and become a stronger motivation than the ambition and curiosity of the undergraduates.

The likelihood of not having a spouse with children to support during medical school is high, therefore one could assume that these private obligations are more common in the postgraduate cohort. Other studies confirm that non-traditional, more mature students have more life commitments than younger students [33]. Our cohorts contradict this assumption and show that private obligations whether towards the family or employer are circumstantial and can be witnessed at any time, thus researchers should be aware of these struggles and consider such obligations in their decision-making regarding when to undertake research.

Our data shows that educational supervisors almost always delegate their responsibility to mentors. While these mentors could successfully fulfil the duties of the supervisors, inexperience, lack of educational training, disinterest, and lack of commitment are factors that disturb this mentoring and default the supervision responsibilities back to the educational supervisors who are either uninterested or do not have time to guide the doctoral candidates, both undergraduate and postgraduate. Students’ perception of research and required skills has been shown to be unrealistic [34], which might explain why these were lacking. This observation is supported by another work that showed a mismatch between supervisors’ and students’ assessment of reasons to abandon doctorates. Moreover, it was shown that both supervisors and students feel unprepared for their respective roles during doctoral projects [7]. Supervision and mentorship could significantly improve if they were tailored to accommodate the researcher’s educational needs and matched the teacher-student stage of learning based on the Grow’s model [25]. Mentors have another impact on the future of doctoral students. Students are more likely to specialise and train in a specialty where they experienced their role models [35]. Thus, mentors and educators should aspire to embody good role models to secure continuous inflow of interested juniors into their field. Some universities acknowledged the importance of this communicative collaboration between the students and supervisors, and developed guidelines to nurture it and thereby improved their completion rate [8].

The fact that ambition and scientific curiosity were not motivating factors for the postgraduate cohort, sustaining motivation to keep working on the project was a struggle for most of them. This meant that this cohort was more likely to procrastinate and spend time away from finishing their research. Some of them genuinely did not enjoy research which increased the struggle to maintain their momentum throughout the process. This contradiction between their aversion to research and their need to complete their identity accompanied them for years. A longer time spent in doing a doctoral research is associated with a higher likelihood of abandoning the project [9]. The ambivalence throughout this period was a source of continuous guilt for our cohort that prevented them from undertaking joyful activities while they were procrastinating. Failing to enjoy life, work, and training increased their feeling of guilt which in turn amplified their negative attitude towards research and resulted in a vicious circle the was difficult to break. We think that this cohort might benefit from the introduction of wellness education programmes and targeted counselling as measures to improve learner success [36].

The myriads of challenges and difficulties facing doctoral candidates might not be directly caused by the timing of the doctorates in relation to graduation, rather the developmental stage of the learner. Their circumstances and motivations mould the profile of the individual’s struggle that educators need to anticipate in order to effectively guide their learners through one of the most important undertakings of their professional career. The main two reasons for abandoning medical doctorates are professional work-related commitments and suboptimal supervisory support [9].

The introduction of a doctorate into the duties and stressors of speciality training places medics in an especially vulnerable position and increases their susceptibility to burnout which raises the question of whether they are supported differently from the undergraduate cohort [37].

It seems that a personal relationship with the mentor in a postgraduate setting is important or at least more likely. This could be an expected observation from a developmental standpoint as postgraduate doctorates are undertaken by a population which is older than undergraduate students and more likely to be friends with colleagues who are active researchers that function as mentors. The interpersonal relationships in this context play a more meaningful role than for the undergraduates because impressing or not disappointing the mentor would be a more important motivation for finishing the projects.

Could it be that family and friends perceive undergraduates as especially vulnerable or as young individuals who require help and support until they graduate? Do graduating and moving to a working environment change how we are perceived by the world and give us the appearance of a hardened shell, thereby decreasing the readiness of others to offer their help? Or do we gain self-sufficiency with age that we become unwilling to ask for help? These questions might not be answerable by the data of this project, but they are valuable thoughts worthy of reflection. One’s perception of own risks and vulnerability might affect one’s behaviour which consequently affects recognition and readiness from others to offer assistance [38].

Research groups resemble communities of practice where the doctorate candidates join this community as newcomers, participate and interact with the established team members to gain skills and knowledge and move towards a more meaningful or established position [39]. This perspective on the role of peers and research teams as a community of practice highlights the interpersonal interactions as a source of support. Instructional communication research shows that different interpersonal communication of the learners at various levels increases their resilience and leads to more effective academic strategies [40]. Doctoral researchers should be protected by providing a state of wellness for them. This can be trained and taught through various initiatives which vary from providing wellness training, psychological training, mentorship, wellness curricula, to encouraging reflective practice. Implementing these initiatives especially in speciality training, where the risk of stressors could be higher, empowers the trainees to build the required resilience and improves the utilisation of wellness tools [41]. The balance between the struggles and support of doctoral candidates is the key during the conduct of the projects that governs whether doctorates fail or succeed as shown in Fig. 1.

The positive impact of doctorates undeniably outweighs the negative aspects of doing research regardless of whether it was done during medical school or speciality training.

The undergraduate cohort significantly benefited from research on professional and personal levels. The career progression could be attributed to either the general skills gained from interacting with research and improving intellect, or to topic-specific knowledge which gave advantage over novices. Undergraduate research promotes interesting careers that combine both research and patient care [42].

The difference between the professional outcomes of both cohorts could be interpreted as higher versatility of the undergraduate cohort. This cohort started their research with ambition, curiosity, and more numerous motivations so they might be more impressionable and susceptible to allow themselves to be captured by the magic of research. They could be a more elastic dough that can be moulded into future researchers. If this holds true, then it is very important to promote undergraduate doctorates in order to cultivate future researchers and secure the future of medical developments. We would recommend every undergraduate to apply for a free semester while they conduct a doctorate. It is essential to protect the physical and mental health of our future medical force and sparing them post-traumatic distress does not only reduce their risk of burnout, but also increases the likelihood of their pursuit of academic and research careers thereby progressing medical knowledge and improving healthcare.

The diversity of the study participants in terms of age, gender, speciality, and university could be considered a strength. The depth of the interviews which were based on a theory-driven interview guide is another strength that increases the credibility of our findings. While the small sample size might be considered a weakness of this work, the quality and richness of the acquired data increase the confidence in the emerged themes despite the lack of evidence for saturation. Moreover, the relative recency of the postgraduate doctorates compared the undergraduates in this cohort, as evident from Table 1, might have affected the recollection of the undergraduate cohort and this could have led to distorted reporting.

## Conclusion

Postgraduate doctorates are neither inherently better nor worse than undergraduate doctorates. Nevertheless, it seems that the timing of the doctorate affects the experience of German researchers. It is reductionist to claim that the experience of all undergraduate or postgraduate researchers is the same because experience is a multifaceted circumstantial state, and the circumstances of every researcher are specific and context dependent. This study identified four important themes that shape the research experience and are partly affected by the timing of the doctorates. Developing a professional identity is the unanimous motivation for doctorates among German medics but undergraduates are additionally motivated by ambition and scientific curiosity. Therefore, promoting undergraduate research as an educational practice could foster future scientists and research enthusiasts. Difficulties facing doctoral candidates are not directly related to the timing of research in relation to graduation, rather to their individual developmental stage. Researchers and educators should be informed about these numerous struggles for developing mitigation plans. Mentors, friends and family as well as peers and research groups are essential source of support to overcome research difficulties. These findings impact one’s mentoring style and could improve their role as a supervisor for doctoral candidates. Undergraduate doctorates are beneficial on both professional and personal levels whereas the postgraduate doctorate produces personal benefits. The benefits of undergraduate doctorates come at the cost of physical and psychological stress that prompts the recommendation of free semester application. We do not claim that this study is exhaustive or that these findings are conclusive and representative of the entire contingent of German undergraduate and postgraduate doctoral candidates, but it provides insight into interesting themes that can be further explored in future work.

## Supplementary Information

Supplementary Material 1.

## Data Availability

The datasets used and/or analysed during the current study are available from the corresponding author on reasonable request.
