# Supplementary material for: The impact of doctorate-timing, whether undergraduate or postgraduate, on the experience of German doctors
Source: BMC Med Educ. 2026 Feb 19;26:341. doi: 10.1186/s12909-026-08849-w (PMC12930819; doi:10.1186/s12909-026-08849-w)
Supplement: Supplementary file 1 — Supplementary Material 1. [file 12909_2026_8849_MOESM1_ESM.docx]

**Interview guide**

**The start**

- A welcome and an introduction of the interviewer
- Describing the course of the interview
- Agreeing on the planned interview duration (45-60 minutes)
- Information regarding the use and release of the collected data
- Obtaining informed consent
- Are there any questions before we start?

**The beginning**

A simple introduction with some simple questions "ice breakers".

- How long have you been working in this department?
- How long has it been since you did your doctorate?
- What was the title of your thesis?
- Where did you do your research?
- Have you tried to start a research project more than once?

**The main part**

Various open and closed questions selected for providing the key answers to the research question.

- When did you start and finish your research project in relation to your UG studies?
- Could you tell how you chose your research topic and how it was initiated?
- What reasons, in your opinion, motivated you to start your doctorate?
- What do you think of the doctoral contract with your supervisor?
- Can you describe what your working relationship with your supervisor was like before and during the project?
- What difficulties, if any, did you encounter in the course of your doctorate?
- Did you get help to resolve these difficulties? In what form and from whom?
- What was the end result of your project? How satisfied are you with it?
- Looking back, how prepared were you for your research and why?
- Do you have long-lasting general or subject-specific learning outcomes from your doctorate that help you in everyday worklife?
- If you could rewrite the past, what would you change about your doctorate experience?
- Would you like to say anything else about the subject?

**Review**

- After all questions and topics have been covered, the interview is briefly summarized.
- Thanking the interviewee for their time and participation in this work.

**Outlook**

- Sharing what will happen next with the answers and whether and when results of this research will be published.
